
JOURNAL INFORMATION
==============================
NLM Title Abbreviation: Innov Surg Sci
Journal ID: Innov Surg Sci
NLM Journal ID: 101708165
Journal ID: iss

Innovative Surgical Sciences

EISSN: 2364-7485
Publisher: De Gruyter

ARTICLE INFORMATION
==============================
PMCID: PMC11917181
PMID: 40110101
DOI: 10.1515/iss-2025-2007
Article ID: iss-2025-2007
Article version: 1
Subjects: Article

Abstracts DGNC

Electronic publication date: 2025 Mar 19
Publication date: 2025 Mar
Volume: 10
Issue: Suppl1
First page: 371
Last page: 375
Copyright: © 2025 by Walter de Gruyter Berlin/Boston
Copyright year: 2025
Copyright holder: De Gruyter
License: This work is licensed under the Creative Commons Attribution 4.0 International License.
License URL: https://creativecommons.org/licenses/by/4.0/

Page count: 5

==============================

Abstracts – DCK 2025 – 142nd Congress of the German Society of Surgery – Munich, March 26–28 • DOI 10.1515/iss-2025-2007
Innov Surg Sci 2025; 10 (Special Suppl 1): s371–s375

**Augmented-Reality assisted placement of ventricular drains in a head model**
ID: 397

**Kategorie: DGNC - Anwendung der KI in der Neurochirurgie**

Max Schneider[1], Christian-Peter Kunz[2], Franziska Mathis-Ullrich[2], C. Rainer Wirtz[1], Andrej Pala[3], Michal Hlavac[3]

[1]*Universitätsklinikum Ulm, Klinik für Neurochirurgie, Ulm, Deutschland*, [2]*Friedrich-Alexander-Universität Erlangen-Nürnberg, Surgical Planning and Robotic Cognition (SPARC), Dep. Artificial Intelligence in Biomedical Engineering (AIBE), Erlangen, Deutschland*, [3]*Bezirkskrankenhaus Günzburg, Klinik für Neurochirurgie, Günzburg, Deutschland*

**Background:**
Augmented reality (AR) headsets are used in medicine for experimental and educational applications. They can superimpose 3D holograms derived from imaging over body parts, e.g. the anatomy of the skull over a head. Ventriculostomies (VST) with external ventricular drains (EVDs) are usually performed using anatomical landmarks, which leads to unfavorable positions in 30-40% of cases. The presented project aims to improve this rate through holographic visualization of the cranial anatomy, entry point and target area for EVD placement. Commercially available AR glasses are used as the only active component in order to minimize disruption to the workflow of this standard procedure.

**Materials and methods:**
A modular head model and automated segmentation algorithm were developed. The precision of the setup was confirmed before a series of VSTs were performed. Routine computed tomography (CT) scans of the head models were used. Entry point and target of EVD placement are automatically computed and could then be verified by the surgeon using AR vision. A passive marker system is attached to the head model. An initial manual registration is required before the AR goggles can track the marker (this was bypassed using pre-registered data in order to streamline the test procedure). A Microsoft HoloLens Gen. 1 served as the hardware platform.
Eleven surgeons performed two VST per head and placed a total of 110 EVDs under AR-guidance. Some weeks later, the procedure was repeated without AR (free hand procedure as per clinical standard). Four of the surgeons performed two additional AR-based series of VST to test for a possible learning curve. Each head model was equipped with an individually molded pair of ventricles before filling with agarose gel. Regular anatomy as well as anomalies such as midline shifts or deformities were included. The EVD catheters were left in place and CT scans were performed to verify the accuracy of placement.

**Results:**
The system functioned reliably and showed stable performance. The overall rate of successful freehand VST was 72.7% vs. 68.8% AR-based. The subgroup with repeated puncture series showed a significant improvement in precision and success rate, going from initially 65% to 95%. The surgeons rated the setup suitable for the test procedure and the overall feedback was positive, however some experienced visual issues when trying to keep hologram as well as head model fixated. General concerns were expressed regarding the reliability of the AR-headset hardware during surgical procedures. For most surgeons, it was the first encounter with AR-headset technology.

**Conclusion:**
AR-guided placement of EVDs in head models with different ventricular anatomy proved to be feasible. The precision of the AR-guided EVDs was satisfactory. Initial results did not differ significantly from success-rate of the free hand procedure. However, repeated use of the AR setup led to a significant improvement of results in subgroup.
The results obtained in this project are promising. The learning curve with the proposed system appears to be steep.
Several more developmental steps have to be taken before an application in humans can be considered safe. In the future, AR-headset based holograms have the potential to be well accepted in clinical application and serve as a compact navigational help inside and outside the operating room.

Open Access. © 2025 The Author(s), published by De Gruyter. This work is licensed under the Creative Commons Attribution 4.0 International License.

**Opportunistic osteoporosis screening in intraoperative CT can accurately identify patients with low volumetric bone mineral density and osteoporosis during spine surgery**
ID: 640

**Kategorie: DGNC - Anwendung der KI in der Neurochirurgie**

Sebastian Rühling[1], Moritz R. Hernandez Petzsche[1], Maximilian T. Löffler[1 2], Nico Sollmann[1 3 4], Thomas Baum[1], Jannis Bodden[1], Julian Schwarting[1], Anastasia Janas[5], Nicole Lange[6], Kaywan Aftahy[6], Maria Wostrack[6], Claus Zimmer[1], Jan S. Kirschke[1]

[1]*Department of Diagnostic and Interventional Neuroradiology, TUM School of Medicine and Health, Klinikum rechts der Isar, Technical University of Munich, Munich, Deutschland*, [2]*Department of Diagnostic and Interventional Radiology, University Medical Center Freiburg, Freiburg, Deutschland*, [3]*TUM-Neuroimaging Center, Klinikum rechts der Isar, Technical University of Munich, Munich, Deutschland*, [4]*Department of Diagnostic and Interventional Radiology, University Hospital Ulm, Ulm, Deutschland*, [5]*Department of Neurosurgery, Charité-Universitätsmedizin Berlin (Corporate Member of Freie Universität Berlin and Humboldt-Universität zu Berlin), Berlin, Deutschland*, [6]*Department of Neurosurgery, TUM School of Medicine and Health, Klinikum rechts der Isar, Technical University of Munich, Munich, Deutschland*

**Background:**
Osteoporosis is prevalent among elderly patients undergoing spine surgery, increasing postoperative complication risks. Although dual-energy X-ray absorptiometry (DXA) is the standard diagnostic tool, its limitations and inconsistent preoperative use often leave bone quality undocumented. Opportunistic volumetric bone mineral density (vBMD) assessment using routine computed tomography (CT) data is a promising alternative. However, preoperative CT scans may be unavailable or incomplete. In such cases, intraoperative multi-detector CT (MDCT) scans used for navigation could provide vBMD measurements. Integrating vBMD assessments intraoperatively could allow surgeons to tailor procedures based on immediate bone quality information. Therefore, the purpose of this study was to evaluate the accuracy of opportunistic vBMD measurements in intraoperative MDCT scans, using preoperative MDCT as the reference.

**Materials and methods:**
This retrospective single-center study included 105 patients (mean age 73 ± 12.6 years; 53 women) who underwent spine surgery for various indications between January 2019 and September 2023. All patients had preoperative MDCT scans with or without intravenous contrast and unenhanced intraoperative MDCT scans. VBMD of thoracolumbar vertebrae was automatically extracted using a convolutional neural network (CNN)-based framework with asynchronous calibration and contrast-phase correction. Two experienced radiologists independently reviewed all MDCT scans for measurable vertebrae prior to any vBMD measurements. Vertebrae affected by artifacts, fractures, or severe degeneration were excluded from analysis. Root-mean-square errors (RMSEs) for associations between paired vertebrae from preoperative and intraoperative vBMD values were calculated using linear regression models. Mean bias and 95% limits of agreement (LOA) were assessed using Bland–Altman plots.

**Results:**
Strong associations between preoperative and intraoperative vBMD values were observed in the thoracic ($R^2$=0.94) and lumbar spine ($R^2$=0.96). Intraoperative vBMD values showed high accuracy in reference to preoperative measurements with a mean bias of -1.3 mg/cm$^3$ for the thoracic spine (LOA: -18.7 to 16.1 mg/cm$^3$) and -3.0 mg/cm$^3$ for the lumbar spine (LOA: -17.4 to 11.3 mg/cm$^3$). RMSEs between preoperative and intraoperative vBMD values slightly increased for contrast-enhanced scans ($RMSE_{thoracic}$: 8.42 vs. 10.1 mg/cm$^3$; $RMSE_{lumbar}$: 7.75 vs. 8.87 mg/cm$^3$). Intraoperative vBMD assessments showed a sensitivity of 100% and a specificity of 95.3% for diagnosing osteoporosis.

**Conclusion:**
Opportunistic osteoporosis screening using intraoperative MDCT data shows high accuracy when compared to preoperative MDCT scans. This approach allows for the identification of patients with low bone mass or osteoporosis during surgery, enabling the use of tailored surgical techniques to reduce postoperative complications. However, the need for manual selection of measurable vertebrae remains a limitation that is to be addressed in consequent studies.

### Artificial intelligence-based intraoperative diagnostics of Glioblastoma and Lymphoma using label-free stimulated Raman histology

ID: 784

**Kategorie: DGNC - Anwendung der KI in der Neurochirurgie**

Jakob Straehle[1 2], Nicolas Neidert[1 2], Pierre Scheffler[1 2], Peter Reinacher[3], Daniel Erny[2 4], Roland Roelz[1], Marco Prinz[2 4], Volker Coenen[2 3], Oliver Schnell[1 5], Dieter Heiland[1 2 5], Jürgen Beck[1 2]

[1]*Klinik für Neurochirurgie, Universitätsklinikum Freiburg, Freiburg, Deutschland*, [2]*Center for Advanced Surgical Tissue Analysis, Universitätsklinikum Freiburg, Freiburg, Deutschland*, [3]*Abteilung Stereotaktische und Funktionelle Neurochirurgie, Universitätsklinikum Freiburg, Freiburg, Deutschland*, [4]*Institut für Neuropathologie, Universitätsklinikum Freiburg, Freiburg, Deutschland*, [5]*Klinik für Neurochirurgie, Universitätsklinikum Erlangen, Erlangen, Deutschland*

**Background:**
The optimal management of patients with glioblastoma (GBM) and central nervous system lymphoma (CNSL) requires the selection of treatment strategies based on a histopathological and molecular diagnosis. While medical treatment is the first-line therapy for CNSL, resection is the therapy of choice in GBM. For patients undergoing resection of glioblastoma it was recently shown that the benefit from a gross-total resection was dependent on the epigenetically defined glioblastoma subclass. We developed two artificial intelligence (AI) based prediction models applied to intraoperative label-free stimulated Raman histology (SRH) images. The first model is a classifier to distinguish CNSL from GBM in (stereotactic) tumor biopsies in order to assist in early patient selection. The second model predicts the epigenetic molecular subclass of glioblastoma resection samples to provide a key element for the future development and application of molecular stratified resection strategies.

**Materials and methods:**
Intraoperative stimulated Raman scattering imaging at wavenumber 2845 cm–1 and 2940 cm–1 was performed using the NIO System (Invenio Imaging). SRH images had a pixel size of 467 nm resulting in an imaging time of ~90 s for a 1.7 x 1.8 mm field of view. For the first model, SRH was performed on stereotactic biopsies (30% emergency cases) from patients with suspected CNSL on MR-imaging (n=40 CNSL and n=31 GBM). SRH images were divided into patches (224 x 224 pixels) and used to train and validate a neural network based on the CTransPath architecture. For the second model – the intraoperative molecular stratifier - intraoperative SRH was performed on tissue samples from 66 patients with primary (73%) and recurrent (27%) glioblastoma undergoing either resection (n=63) or biopsy (n=3). DNA was extracted and analyzed for genome-wide DNA methylation patterns using the Illumina EPIC (850k) array. Assessment of epigenetic subclass was performed using the brain tumor classifier (version 12.8) of the DKFZ Heidelberg (42% MES, 12% RTK 1, 42% RTK 2 and 4% PNC). Epigenetic subclass prediction from SRH images was performed by an attention-based graph-neural network (5 attention heads) coupled by a ResNet18 encoder for node feature extraction.

**Results:**
In the first model, the classifier demonstrated an accuracy of 92.5% in distinguishing between lymphoma and glioblastoma. Applied to the test set it showed a sensitivity of 84.2% and a specificity of 100% in the detection of CNSL. In the second model, the 10-fold cross validation demonstrated an overall accuracy of 87% to predict the epigenetic subclasses of glioblastoma. Using graph attention and integrated gradients, we identified image pattern and neighbourhood relationships associated with the different subclasses. The highest overlap was observed between the RTK II and MES subgroups. Processing times ranged from 5.6 to 8.4 minutes from biopsy acquisition to subtype prediction, which is significantly faster than conventional molecular analyzes requiring several days of processing time. This rapid turnaround time is essential for intraoperative decision-making, as it would enable neurosurgeons to adapt their surgical strategies based on the predicted epigenetic subtype in real-time.

**Conclusion:**
We present two AI-based prediction models based on intraoperative stimulated Raman imaging to first assist in rapid patient selection in the case of stereotactic/intraoperative biopsies and second to predict epigenetic molecular subtypes during glioblastoma resection. The accuracy of the AI-based classifier of 92.5% in distinguishing between lymphoma and glioblastoma is on a par with standard intraoperative histopathological analysis. The accuracy of the AI-based prediction of epigenetic subclasses of 87 % even surpasses recently published classification results based on intraoperative sequencing. SRH imaging and AI-based image classification are fast and easily applicable during routine neurosurgical procedures and are therefore promising methods for intraoperative molecular diagnostics.

Abstracts – DCK 2025 – 142nd Congress of the German Society of Surgery – Munich, March 26–28 • DOI 10.1515/iss-2025-2007
Innov Surg Sci 2025; 10 (Special Suppl 1): s371–s375

### Stimulated Raman Histology for Rapid Intraoperative Diagnosis Prediction in Spinal Tumor Surgery

ID: 371

**Kategorie: DGNC - Spinale Onkologie**

David Reinecke[1], Nina Müller[1], Anna-Katharina Meissner[1], Gina Fürtjes[1], Lili Leyer[1], Abdulkader Al-Shugri[2], Tobias Blau[3], Roland Goldbrunner[1], Niklas von Spreckelsen[1], Volker Neuschmelting[1]

[1]*Klinik für Allgemeine Neurochirurgie, Köln, Deutschland*, [2]*Institut für Neuropathologie, Köln, Deutschland*, [3]*Institut für Neuropathologie, Essen, Deutschland*

**Background:**

Intraoperative decision-making during spinal tumor surgery is often dependent on pathologic diagnosis by fresh frozen rapid section histopathology. Recently, stimulated Raman scattering (SRS) microscopy has been introduced as alternative approach to generate digital hematoxylin-and-eosin-stained-like images (Stimulated Raman histology, SRH) within a few minutes from squeezed fresh tumor samples intraoperatively. The images can then be analysed by a pathologist or a machine learning algorithm. So far algorithm based analysis of SRH-Images has only been developed and validated for brain tumors[1], but not for spinal tumors. In this study, we aimed to (1) analyze whether previously published algorithms for brain tumour diagnostics could accurately differentiate corresponding spinal tumor manifestations. (2) If not sufficiently accurate, we aimed to train an develop a new AI-based analysis algorithm to outperform the existing tools in particular for spinal tumor applications.

**Materials and methods:**

Tissue samples were obtained intraoperatively from the lesions itself and a small unprocessed specimen sample was scanned with a field-of view of 3x3 mm in a fiberlaser SRS microscope. SRH Images were then analyzed by a previously published AI algorithm[1] for prediction of the histologic brain tumor classes. The samples were classified into 13 histological categories: ependymoma, malignant glioma, diffuse low grade glioma, lymphoma, medulloblastoma, meningioma, metastasis, pilocytic astrocytoma, pituitary adenoma, gliosis, white matter and grey matter and non-diagnostic. The new spinal tumor specific AI analysis tool was developed on the same data set using a patch based bootstrap your own latent (BYOL) and cross-entropy loss learning (CE) supervised learning method.

**Results:**

We evaluated more than 200 SRH images from 99 patients with spinal lesions undergoing tumor resection. Of these 50.5% (n=50) had dural involvement. 22 of these (44%) were meningiomas, 22 (44%) metastases, 4 (8%) Lymphoma, 1 (2%) chloroma and 1 (2%) Osteosarcoma. The brain tumor AI analysis tool correctly classified all 22 spinal meningiomas as such. Prediction of all other corresponding spinal tumors classes did not perform well. We then successfully trained and developed a new spinal tumor specific AI based analysis tool that outperformed the existing brain prediction tumor tool in all spinal tumor classes except meningioma (already previous high accuracy). Rare entities such as chloroma, sarcoma and osteoblastoma could not yet be trained correctly since too low in incidence and not well represented in the existing training data set.

**Conclusion:**

Intraoperative SRH-Imaging enables digital H+E like images within a few minutes that are readily available for pathological review or subject to observer independent AI based analyses. However, existing AI algorithms developed for classification of brain tumors[1] does not perform well in the prediction of corresponding spinal tumor entities. To reliably differentiate these tumors and accelerate intraoperative decision-making an AI algorithm specifically trained for spinal tumors is needed and could successfully been developed differentiating the most common spinal tumor classes with high accuracy. Further developmental effort is needed for a broad based application of this innovative and promising technique in spinal oncology surgery.

[1]Hollon, Todd C. et al., *Nature Medicine* 26.1 (2020): 52-58.
